# Hepatitis B and C among Patients Infected with Human Immunodeficiency Virus in Isfahan, Iran: Seroprevalence and Associated Factors

**Published:** 2010-09-01

**Authors:** Behrooz Ataei, Katayoon Tayeri, Nazila Kassaian, Ziba Farajzadegan, Anahita Babak

**Affiliations:** 1 Isfahan Infectious Diseases and Tropical Medicine Research Center, Isfahan University of Medical Sciences, Isfahan, Iran; 2 Triangular Clinic, Isfahan University of Medical Sciences, Isfahan, Iran

**Keywords:** HBV, HCV, HIV, Coinfection, Iran

## Abstract

**Background and Aims:**

Patients with human immunodeficiency virus (HIV) are also likely to be at risk for other infectious pathogens including hepatitis B(HBV) and C(HCV) viruses, which complicate the clinical course, management, and therapy. The literature on the prevalence of HBV/HCV coinfection with HIV in Iran is sparse. Hence this study was conducted to investigate this coinfection pattern and its risk factors in Isfahan, Iran.

**Methods:**

All of the HIV-infected patients attending clinics for acquired immune deficiency syndrome (AIDS) research and  education in Isfahan province during the period of May 1998 through April 2007 were included in this cross-sectional study. After giving their informed consent, the patients were screened for hepatitis B surface antigen (HBsAg) and antibodies to hepatitis C virus (anti-HCV), and anti-HCV-positive cases were confirmed with the RIBA test. The demographic data and information about risk behaviors were collected as well. Multivariate logistic regression was used to identify independent risk factors for HBV and HCV.

**Results:**

The subjects included 130 patients (128 males and 2 females) with a mean age of 50.23 ± 8.81 years. Most of the subjects were unemployed (61.5%) and single (56.2%). A history of imprisonment, ,intravenous drug abuse, and high-risk sexual activity were reported by 83.7%, 83.5%, and 48% of the subjects, respectively. Coinfection with hepatitis viruses was observed in 78.5% of the subjects. Low levels of education, a history of imprisonment, and youth were the main risk factors for HCV/HIV coinfection (OR = 196, 114, and 0.9 respectively).

**Conclusions:**

Our study showed that there is a high prevalence rate of HCV/HIV coinfection in Isfahan, Iran, with the major risk factor being a history of imprisonment.

## Introduction

Patients with human immunodeficiency virus  (HIV) are also likely to be at risk for other infectious pathogens. Coinfection with HIV and hepatitis B virus (HBV) and/or hepatitis C virus (HCV) is common because all of these diseases are spread by similar routes of viral transmission. Of late, liver diseases due to chronic hepatitis B and C infection are becoming a leading cause of death and decrease life expectancy among persons with HIV infections worldwide [1]. On the other hand, viral hepatitis complicates the clinical course and management, and may also adversely affect therapy, for HIV infection [2]. The evidence has shown that highly active antiretroviral therapy (HAART) effectively prevents AIDS, but this regimen does not suppress HCV [3].

For these reasons, in the United States and Europe,  the screening of all HIV-infected persons for HBV and HCV is recommended [4]. Various international studies have been conducted to demonstrate the rate of coinfection with either HCV or HBV, and the results naturally vary according to country and subpopulation.[3]. Indeed, the prevalence varies, depending on the patient's risk factors for HIV acquisition [1]. Few studies have focused on the prevalence of coinfection with HIV and HBV and/or HCV in Iran. Hence, in this study we investigated the coinfection pattern of HBV and HCV among HIVinfected patients and their risk factors in Isfahan, Iran to support the implementation of future strategies to improve national and international monitoring of this problem.

## Materials and Methods

This cross-sectional study was approved by  the Ethical Committee of Isfahan University of Medical Sciences One hundred and thirty subjects were recruited from clinics for AIDS research and education and triangular clinics in Isfahan province (a province in central Iran) from May 1998 through April 2007. HIV confirmed by means of the enzymelinked immunosorbent assay (ELISA) (Diapro-Italy) and Western Blot (Immunogenetic, Germany) tests.

he triangular clinics in Iran make use of dedicated staff to deliver services such as harm reduction to drug abusers, to people who want to receive HIV counseling and testing, to people living with HIV/AIDS and their families, to high-risk populations, to patients with STDs, and to persons who have been exposed to potentially contaminated bodily fluids. These clinics provide services both for prevention and care. Participation in the study was on a voluntary basis, and after obtaining informed consent, 5cc of venous blood was taken from each subject. Diagnosis of HBV and HCV infection was made using the ELISA metho anti-HCV kit (Dia-pro Diagnostic Bioprobes s.r.l., third generation, Italy) and the HBsAg kit (Diapro Diagnostic Bioprobes s.r.l. -Italy) respectively. The anti-HCV positive subjects were confirmed with a RIBA test (Immunogenetic, Germany). Among the subjects, one patient, a responsible physician, recorded sociodemographic information including age, marital status, employment status, history of imprisonment, and high-risk behaviors (intravenous drug abuse and high-risk sexual activities).

Descriptive statistics are presented in Table 1 with the means ± standard deviation or proportions for continuous or categorical variables, respectively. The normal distribution of continuous variables was validated by a Kolmogorov-Smirnov test, and a multivariate logistic regression model was used to obtain the risk factors for HBV and HCV. Data analyses were carried out with the Statistical Package for Social Sciences software (SPSS, version 15). P-values < 0.05 were considered statistically significant.

**Table 1:** Demographic Characteristics of the HIVinfected Subjects (n = 130).

	**Demographic Characteristics**	**Number**	**Percent**
**Gender**	Male	128	98.5
	Female	2	1.5
**Occupation**	Yes	50	38.5
	No	80	61.5
**Education**	Illiterate	9	7
	Elementary school	52	40.3
	Secondary school	33	25.6
	High school	10	7.8
	Diploma	22	17.1
	Academic education	3	2.3
**Marital status**	Not married	73	56.2
	Married	37	28.5
	Divorced	18	13.8
	Widow	2	1.5
**Prison History**	Yes	108	83.7
	No	21	16.3

## Results

The subjects included 128 male and 2 females. The mean age (with the normal distribution of age validated by a Kolmogorov-Smirnov test) was 50.23 ± 8.81 years. The main demographic characteristics in the participants are presented in table 1.One hundred and six (83.5%) of the subjects were intravenous drug abusers, and 61 (48%) had engaged in highrisk sexual behavior. Forty subjects from these two groups (31.5%) showed both risk factors. None of the subjects admitted to homosexuality..Most types of sexual behavior besides that within the marital state were considered unsafe, Of the 130 HIV-infected patients, 102 (78.5%) were coinfected with one or both of the hepatitis viruses.

According to the laboratory tests, 15 subjects (11.5%) were HBsAg positive, 100 (77%) were anti- HCV positive, and 12 subjects (9.2%) from these two groups were positive for both. After entering the variables into the multivariate logistic regression model, none of them showed statistical significance for coinfection with HBV or HBV/HCV  but among the HCV/HIV coinfected subjects, the predominant observed risk factors were low education, a history of imprisonment and relative youthfulness (Table 2).

**Table 2:** HIV/HCV Coinfection risk Factors in the Logistic Regression Model (n = 130).

	**Variables**	**No adjusted OR(95%CI)**	**P-value**	**Adjusted OR (95% CI)**	**P-value**
**Education (a)**	Illiterate	16 (0.66-383)	0.87	196 (2.4-16065)	0.019^e^
	Elementary school	8.4 (0.69-102)	0.095	76 (2.2-2685)	0.017^e^
	Secondary school	4.6 (0.37-56.7)	0.23	30.7 (0.85-1115.4)	0.062
	High school	18 (0.75-427)	0.07	164.3 (1.7-15768)	0.028^e^
	Diploma	5.3 (0.4-70.2)	0.2	75 (1.5-3788.5)	0.031^e^
**Being in prison (b)**		23.4 (7.3-74.5)	0.000^e^	114.35 (4-3226.5)	0.005
**Occupation (c)**		2.65 (1.2-6.1)	0.02^e^	1.9 (0.51-7)	0.33
**Age**		0.96 (0.9-1.01)	0.12	0.9 (0.83-0.99)	0.03^e^
**High-risk sexual behavior (b)**		0.22 (0.08-0.58)	0.002^e^	0.74 (0.2-2.7)	0.65
**IV drug abuse (b)**		13 (4.5-38.2)	0.000^e^	0.59 (0.04-8.7)	0.7
**Marital Status (d)**	Widow	0.6 (0.03-10.52)	0.73	0.8 (0.001-1046.9)	0.95
	Divorce	4.8 (0.97-24.4)	0.054	2.5 (9.21-30.7)	0.46
	Never-married	2.8 (1.15-6.9)	0.02^e^	0.5 (0.07-3.4)	0.48

^a^ Statistically significant

^b^ Reference category: Academic education

^c^ Reference Category: No

^d^ Reference Category: Yes

^e^ Reference Category: Married

^f^

## Discussion

This study indicated that viral hepatitis coinfection is common in HIV-infected patients in Isfahan, Iran (78.5%). According to the evidence, since HIV, HBV, and HCV are transmitted in similar ways, many people are infected with two or even all three of these viruses [5]. It is estimated that the average prevalence of HCV among people living with HIV/AIDS is 40% worldwide [6][7]. Also, it has been shown that chronic HBV infection as identified by the presence of the HBsAg is found in 5–15% of HIV-infected patients globally [2].

Other studies have indicated that HBV infection among those infected with HIV varies from 5–10% in the United States to 20–30% in Asia and parts of Sub-Saharan Africa [1].

In one recent study, the prevalence rates of HBV/ HIV, HCV/HIV, and HBV/HCV/HIV coinfection were 77%, 11.5%, and 9.2%, respectively. Only a few studies have been carried out on HIV/HBV/HCV coinfection in different parts of Iran. For example, in a study of 104 HIV-infected patients in Ahwaz from 2001 to 2003, 44% and 74% were HBsAg- and anti- HCV-positive, respectively [8].

In another study on injecting drug users in Tehran, Iran, the prevalence rates of HBV and HCV among HIV-infected individuals were 77% and 80.6%, respectively [9]. Finally, in a study carried out  on 391 HIV-positive patients in Lorestan province, coinfection with hepatitis viruses was 94.4%, of whom 14.5% were HBsAg positive, 79% were anti-HCV- positive, and 7.9% were both HBsAg and anti-HCV- positive [10]. There is a variety of information from other countries as well.

A study in India revealed that 9% and 2.2% of HIV-infected patients were coinfected with HBV  and HCV, respectively [11]. In a study of Spanish prisoners, the prevalence of HCV/HIV coinfection was 18.8% [12]. In another study in Germany, 9% and 23% of HIV-infected patients were HBV/HIV and HCV/HIV coinfected, respectively [13]. In a study from the USA, the overall estimated HCV prevalence among patients infected with HIV was 16.1% [14]. Finally, in another investigation, this time from Afghanistan, 1.5% of participants were coinfected with HIV and HCV [15].

Our present findings showed that the predominant risk factors for HCV/HIV coinfection were having little education, having a history of imprisonment, and also being relatively young.

The risks of being in a prison environment have been well-documented. Some these risk factors are sharing needles, razors, and tattooing equipment, among others. For these reasons, imprisonment is a key structural factor fuelling outbreaks of HIV and  HCV in a number of countries [16][17][18][19].

The higher prevalence rates of HCV/HIV coinfection in the less educated and younger groups have also been reported in other studies [15][20][21]. This may be due to higher-risk behaviors and lower  awareness in young adults and the less educated. On the other hand, as young people are prone to highrisk behavior, the results seem logical.

Although drug abuse has been the major route of HIV and HCV transmission in Iran [3], after adjusting for variables in multivariate logistic regression analyses, intravenous drug abuse (IVDA) was not included as a significant risk factor for HCV/HIV coinfection in our study. Additionally, in the  present study, no risk factor was detected for HBV or HBV/HCV coinfection. This may have been a result of the low prevalence of HBV and HBV/  HCV coinfection in our subjects or simply the small sample size. Also, because consultants in the clinics for AIDS research and education have been  providing essential education about safe sex (such as the use of prophylactics), it is possible that the sexual transmission of HBV (the most important route for HBV transmission) has had limited scope in these patients.

There are several limitations to the present study that require consideration. First, the study was conducted with patients who attended the triangular and HIV/AIDS clinics and were not studied in a community setting, so we are unable to generalize  our findings outside the study population. Second,we made little use of the polymerase chain reaction (PCR), because of its prohibitive cost, given our limited resources. Some studies have demonstrated that in HIV-infected patients, testing only serological viral markers such as HBsAg, antibodies to hepatits B e antigen (anti-HBe) IgG, and anti-HCV, fails to identify the true prevalence of coinfection with HBV and HCV. The qualitative PCR for HBVDNA and HCVRNA detects coinfection in patients who are negative for serologic markers [20]. Third, because extramarital sexual activity is taboo in our country, it is possible that such behavior.was denied by some of our subjects out of fear of exposure.

## Conclusions

This study shows that there is a high prevalence  of HCV/HIV coinfection in Isfahan, Iran and that being in prison has a considerable effect on this phenomenon. Therefore resources should be allocated for the prevention and treatment of HCV/ HIV coinfection. It would seem advisable to screen for hepatitis viruses in the entire HIV infected population at the earliest possible time. Additionally, the provision of education for high-risk groups and for young people on the prevention and transmission of the infection should be should be considered as well.
